# Caffeoylquinic Acid-Rich Extract of* Aster glehni* F. Schmidt Ameliorates Nonalcoholic Fatty Liver through the Regulation of PPAR*δ* and Adiponectin in ApoE KO Mice

**DOI:** 10.1155/2017/3912567

**Published:** 2017-10-23

**Authors:** Yong-Jik Lee, Yoo-Na Jang, Yoon-Mi Han, Hyun-Min Kim, Jong-Min Jeong, Min Jeoung Son, Chang Bae Jin, Hyoung Ja Kim, Hong Seog Seo

**Affiliations:** ^1^Cardiovascular Center, Korea University, Guro Hospital, 148 Gurodong-ro, Guro-gu, Seoul 08308, Republic of Korea; ^2^Department of Medical Science, Korea University College of Medicine (BK21 Plus KUMS Graduate Program), Main Building 6F Room 655, 73 Inchon-ro (Anam-dong 5-ga), Seongbuk-gu, Seoul 136-705, Republic of Korea; ^3^Molecular Recognition Research Center, Materials and Life Science Research Division, Korea Institute of Science and Technology, Hwarangno 14 Gil 5, Seoul 136-791, Republic of Korea

## Abstract

*Aster glehni *is well known for its therapeutic properties. This study was performed to investigate the effects of* A. glehni* on nonalcoholic fatty liver disease (NAFLD) in atherosclerotic condition, by determining the levels of biomarkers related to lipid metabolism and inflammation in serum, liver, and adipose tissue. Body and abdominal adipose tissue weights and serum triglyceride level decreased in all groups treated with* A. glehni*. Serum adiponectin concentration and protein levels of peroxisome proliferator-activated receptor *δ*, 5′ adenosine monophosphate-activated protein kinase, acetyl-CoA carboxylase, superoxide dismutase, and PPAR*γ* coactivator 1-alpha in liver tissues increased in the groups treated with* A. glehni*. Conversely, protein levels of ATP citrate lyase, fatty acid synthase, tumor necrosis factor *α*, and 3-hydroxy-3-methylglutaryl-CoA reductase and the concentrations of interleukin 6 and reactive oxygen species decreased upon* A. glehni*. Triglyceride concentration in the liver was lower in mice treated with* A. glehni* than in control mice. Lipid accumulation in HepG2 and 3T3-L1 cells decreased upon* A. glehni *treatment; this effect was suppressed in the presence of the PPAR*δ* antagonist, GSK0660. Our findings suggest that* A. glehni *extracts may ameliorate NAFLD through regulation of PPAR*δ*, adiponectin, and the related subgenes.

## 1. Introduction

Nonalcoholic fatty liver disease (NAFLD), characterized by the accumulation of triglyceride in hepatocytes, is one of the most common diseases today. Metabolic disorders such as obesity, diabetes mellitus, and hyperlipidemia are major risk factors for NAFLD and nonalcoholic steatohepatitis (NASH), which is a more severe form of NAFLD [1, 2]. Furthermore, NAFLD can be used as a representative clinical index of hypertension, cardiovascular disease, and diabetic complications [3, 4]. Adiponectin is an adipocytokine consisting of 244 amino acid residues and is specifically and highly expressed in adipose tissues [5]. Its expression is closely related to various metabolic diseases such as obesity, type 2 diabetes, atherosclerosis, and cardiovascular disease [6, 7]. In addition, soybean embryo ameliorates nonalcoholic fatty liver through adiponectin mediated 5′ adenosine monophosphate-activated protein kinase *α* (AMPK *α*) pathway [8]. AMPK normalizes lipid homeostasis through several mechanisms. It downregulates cholesterol and fatty acid syntheses by inactivating the enzymes 3-hydroxy-3-methylglutaryl-CoA reductase (HMGCR) and fatty acid synthase (FASN), respectively. Further, AMPK upregulates fatty acid oxidation by inhibiting acetyl-CoA carboxylase (ACC) [9–11].

Throughout human history, many plants have been consumed not only as food, but also for preventing or even curing certain diseases. For instance,* Aster glehni* has been used in cooking and as traditional medicine for hundreds of years in Korea. In the* Dongui Bogam*, a Korean traditional medical encyclopedia, it is recorded that* A. glehni *exhibits antipyretic and analgesic activities and reduces phlegm and coughing. In addition, various therapeutic functions of* A. glehni* extract such as antiobesity, antioxidation, anti-inflammation, and antiwrinkle activities have been recently reported [12–14]. These studies suggest the potential antiadipogenesis and antiobesity effects of* A. glehni* and its therapeutic potential in treating obesity-related diseases.

Hitherto, there are few studies on the effects of* A. glehni* on metabolic diseases. Because many studies have reported a close correlation between NAFLD and cardiovascular diseases such as hypertension and atherosclerosis, we investigated the effect of* A. glehni* on nonalcoholic fatty liver in atherosclerotic mice and it was conducted with focusing on PPAR*δ*. The present findings can be beneficial in further understanding the role of phytomedicines in treating atherosclerosis and fatty liver disease.

## 2. Materials and Methods

### 2.1. Plant Material

Parboiled and dried* A. glehni *F. Schmidt (family Compositae) were purchased from Ulleung Island, Gyeongsangbuk-do, Korea, in November 2012 and identified by Professor Chang-Soo Yook (Department of Pharmacognosy, Kyung Hee University, Seoul, Korea). Voucher specimens (971-12A-P) were deposited in the herbarium of the Korea Institute of Science and Technology.

### 2.2. Extraction Procedure

Chopped leaves and stem of* A. glehni* (12 kg) were extracted three times with methanol (70 L) at room temperature to give a methanol-soluble extract. The dried extract residue (2.6 kg) was suspended in water and partitioned with ethyl acetate. The ethyl acetate fraction was evaporated under reduced pressure to yield 41.0 g of residue. Organic solvents used in the extraction procedure were purchased from Sigma-Aldrich (St. Louis, MO, USA).

### 2.3. High-Performance Liquid Chromatography (HPLC) Analysis for Ethyl Acetate Extract of* A. glehni*

The ethyl acetate extract of* A. glehni* was analyzed using reverse-phase high-performance liquid chromatography (Waters 1500 Series System), with a 2998 PDA Detector (Waters, Worcester, MA, USA). Separation was performed using a Luna C18 column (5 *μ*m, 250 × 4.6 mm, Phenomenex, Torrance, CA, USA) at 25°C with a sample injection volume of 10 *μ*L. The mobile phase was a gradient of methanol and 1% acetic acid. The following gradient was used: 30% methanol (0 min), 40% methanol (0~10 min), 60% methanol (10~20 min), 80% methanol (20~30 min), and 100% methanol (30~40 min). The flow rate of the mobile phase was 1.0 ml/min. Organic solvents used in HPLC analysis were purchased from Sigma-Aldrich.

### 2.4. Cell Culture

HepG2 cells were cultured in Dulbecco's modified Eagle's medium (DMEM) containing 10% fetal bovine serum (FBS) and 1% antibiotic-antimycotic solution at 37°C in a 5% CO_2_ incubator. The medium was replaced every 48–72 h. HepG2 cells within 95~110 passages were plated at a density of 5 × 10^4^ cells per well in 24-well culture dishes or 1 × 10^6^ cells per well in a 6-well culture plate in DMEM containing 10% FBS and 1% antibiotic-antimycotic solution. Cells were cultured for 24 to 48 h at 37°C in a 5% CO_2_ incubator, and the media were changed to DMEM containing 1% FBS. Thereafter, cells were treated in high fatty acid (0.1 mM palmitate) and high cholesterol (0.2 mM) condition, with* A. glehni *extract (25 *μ*g/mL) and the peroxisome proliferator-activated receptor *δ* (PPAR*δ*) antagonist, GSK0660 (50 *μ*M), for 24 h. HepG2 cells were also treated with dorsomorphin (AMPK antagonist, compound C) of 10 uM for 24 h. 3T3-L1 preadipocytes were cultured in DMEM containing 10% calf serum and 1% antibiotic-antimycotic solution at 37°C in a 5% CO_2_ incubator. The medium was replaced every 48–72 h. 3T3-L1 cells within 8~18 passages were plated at a density of 5 × 10^4^ cells per well in 24-well culture dishes in DMEM containing 10% calf serum and 1% antibiotic-antimycotic solution. When 3T3-L1 cells reached confluence, differentiation media were applied to cells, together with* A. glehni *extract and PPAR*δ* antagonist. The differentiation media contained 0.0125 uM dexamethasone, 12.5 uM 3-isobutyl-1-methylxanthine, 10 *μ*g/mL insulin, and 10% FBS. After differentiation for two days, the media were replaced with insulin media which contained 10 *μ*g/mL insulin and 10% FBS. After incubation in insulin media for 2~4 days, the media were changed to maintenance media which contained only 10% FBS. All the concentrations of chemicals are final treatment concentrations. HepG2 and 3T3-L1 cell lines were purchased from the Korean Cell Line Bank (Seoul, Korea). All reagents for cell culture were purchased from Welgene Inc. (Daegu, Korea). Palmitate, cholesterol, GSK0660, compound C, and reagents for 3T3-L1 cell differentiation were purchased from Sigma-Aldrich.

### 2.5. Animal Study

In total, forty male apolipoprotein E knock-out (ApoE KO) mice were used in this study. Mice (six weeks of age) were adapted to the diet for seven days. Mice were fed diet containing 0.15% cholesterol and were divided into the following four groups: group (1), fed only cholesterol diet; group (2), fed cholesterol diet and 100 mg/kg/day* A. glehni *extract; group (3), fed cholesterol diet and 300 mg/kg/day* A. glehni* extract; and group (4), fed cholesterol diet and 500 mg/kg/day* A. glehni *extract. The extracts of all concentrations were administered through drinking water. All mice were sacrificed after four weeks. The administered amounts of* A. glehni* extracts were determined as follows: first, body weights of animals were estimated every week during whole experimental period, and then the administering amounts of* A. glehni* extracts were calculated on the basis of mean body weight per experimental group and general mean water drinking amount per mouse.

Animal experiments were performed according to the Animal Experiment Ethics Guide of Guro Hospital, Korea University. The experiments complied with the Korea University Animal Research Rules and Regulations, and the protocols were approved by the Korea University Institutional Animal Care and Use Committee (approval number: KUIACUC-2014-40). The ApoE KO mice and 0.15% cholesterol diet were supplied by Doo Yeol Biotech (Seoul, Korea). The cholesterol diet was made by Doo Yeol Biotech adding 0.15% cholesterol to Teklad Global 18% Protein Rodent Diet. Ingredient composition for 0.15% cholesterol diet is described in Table 1.

### 2.6. Estimation of Triglyceride, Total Cholesterol, High-Density Lipoprotein (HDL) Cholesterol, Low-Density Lipoprotein (LDL) Cholesterol, Adiponectin, Interleukin 6 (IL6), and Reactive Oxygen Species (ROS) Concentrations in Serum or the Liver

Concentrations of triglyceride and cholesterols in serum were estimated by using enzymatic colorimetric assay kits (Kyowa Medex Co., Ltd., Tokyo, Japan) in the Department of Laboratory Medicine (Diagnostic Tests), Korea University, Guro Hospital (Seoul, Korea).

Enzymatic colorimetric assay kit (Cayman Chemical, Ann Arbor, MI, USA) was used to measure triglyceride concentrations in the liver tissues, according to the manufacturer's instructions. Sample preparation procedures were as follows. Liver tissue was rinsed in ice-cold PBS to remove excess blood. The minced liver tissue of 300 mg was homogenized in the diluted standard diluent of 2 ml. The extract was centrifuged at 10,000 ×g for 10 min at 4°C, and then the supernatant was transferred to another tube. The supernatant sample was diluted by the ratio of 1 : 5 before use. Standard and sample solutions (10 *μ*L) were added to the wells, and the diluted enzyme buffer solution (150 *μ*L) was added to each well. Afterward, the plate was carefully shaken for a few seconds, covered with a sealing tape, and incubated for 15 min at room temperature. The absorbance at 540 nm was measured using a microplate reader. Triglyceride concentrations in the samples were determined by interpolation from a standard curve prepared using the standard solutions, and expressed in mg/dL.

Adiponectin concentration in serum samples was assayed using an enzyme-linked immunosorbent assay (ELISA) kit that can detect both globular domain and full-length adiponectin from Abcam (Cambridge, UK). Experimental procedures were as follows. The 50 *μ*L of adiponectin standard or sample added to each well of 96 well plates, and after covering wells with a sealing tape, was incubated for 1 hr at room temperature. After washing 5 times with 1x wash buffer of 200 *μ*L, 1x biotinylated adiponectin antibody of 50 *μ*L was added to each well, and it was incubated for 1 hr. After washing 5 times with 1x wash buffer of 200 *μ*L, 1x streptavidin-peroxidase conjugate of 50 *μ*L was added to each well, and it was incubated for 30 min. After washing 5 times with 1x wash buffer of 200 *μ*L, chromogen substrate of 50 *μ*L was added to each well, and it was incubated till the optimal blue color density develops. Stop solution of 50 *μ*L was added to each well, and then, the absorbance was estimated with a microplate reader at a wavelength of 450 nm. Adiponectin concentration in the samples was determined by interpolation from a standard curve prepared with standard samples supplied by the manufacturer, and expressed in ng/mL.

IL6 concentration in the liver tissues was assayed using an enzyme-linked immunosorbent assay (ELISA) kit (Mybiosource, CA, USA). Sample preparation procedures were as follows. Liver tissue was rinsed in ice-cold PBS to remove excess blood. The minced liver tissue of 300 mg was homogenized in the diluted standard diluent of 2 ml. The extract was centrifuged at 5,000 ×g for 5 min at 4°C, and then the supernatant was transferred to another tube. The supernatant sample was diluted by the ratio of 1 : 30 before use. Standard and sample solutions (100 *μ*L) were added to the wells, covered with the plate sealer, and incubated for 2 hr at 37°C. After removing the liquid in each well, detection reagent A of 100 *μ*L was added to each well, and the sealed plate was incubated for 1 hr at 37°C. After aspirating the liquid in each well, wells were washed three times with 1x wash buffer, and detection B of 100 *μ*L was added to each well, and the sealed plate was incubated for 2 hr at 37°C. After aspirating and washing, substrate solution of 90 *μ*L was added to each well, and the sealed plate was incubated for 30 min at 37°C. Stop solution of 50 *μ*L was added to each well, and then, the absorbance was estimated with a microplate reader at a wavelength of 450 nm. IL6 concentration in the samples was determined by interpolation from a standard curve prepared with standard samples supplied by the manufacturer, and expressed in ng/mL.

ROS concentration in the liver tissues was assayed using an enzyme-linked immunosorbent assay (ELISA) kit (Mybiosource, CA, USA). Liver tissue was rinsed in ice-cold PBS to remove excess blood. The minced liver tissue of 300 mg was homogenized in the diluted standard diluent of 2 ml. The extract was centrifuged at 10,000 rpm for 10 min at 4°C, and then the supernatant was transferred to another tube. Experimental procedures were as follows. Standard and sample solutions (100 *μ*L) were added to the wells, covered with the plate sealer, and incubated for 2 hr at 37°C. After removing the liquid in each well, detection reagent A of 100 *μ*L was added to each well, and the sealed plate was incubated for 90 min at 37°C. After aspirating the liquid in each well, wells were washed two times with 1x wash buffer, and biotinylated mouse ROS antibody solution of 100 *μ*L was added to each well, and the sealed plate was incubated for 1 hr at 37°C. After aspirating and washing, enzyme-conjugate solution of 100 *μ*L was added to each well (excepting blank wells), and the sealed plate was incubated for 30 min at 37°C. Color reagent liquid of 100 *μ*L and color reagent C were added to each well sequentially, and the absorbance was estimated with a microplate reader at a wavelength of 450 nm in 10 min. ROS concentration in the samples was determined by interpolation from a standard curve prepared with standard samples supplied by the manufacturer, and expressed in unit/mL.

### 2.7. Semiquantitative Reverse Transcription Polymerase Chain Reaction (RT-PCR)

Total RNA was extracted by the TRIzol reagent® according to manual. Complementary DNA was synthesized by power cDNA synthesis kit from total RNA, and polymerase chain reactions for PPAR*δ*, AMPK*α*1 subunit, interleukin 6 (IL6), tumor necrosis factor *α* (TNF*α*), and *β*-actin were administered with PCR Premix kit. The primer sequences used were as follows: forward 5′-GGCAGAGTTGCTAGGGTTCC-3′ and reward 5′-CAAGGAACACCCCAAGACCT-3′ for mouse PPAR*δ* (PCR product size is 294 bp); forward 5′-CCTGCTTGATGCACACACATGA-3′ and reward 5′-TCATCAAAAGGGAGGGTTCC-3′ for mouse AMPK*α*1 subunit (PCR product size is 213 bp); forward 5′-TTCACAGAGGATACCACTCC-3′ and reward 5′-AAGTGCATCATCGTTGTTCA-3′ for mouse IL6 (PCR product size is 147 bp); forward 5′-CTACTCCTCAGAGCCCCCAG-3′ and reward 5′-CAGGTCACTGTCCCAGCATC-3′ for mouse TNF*α* (PCR product size is 126 bp); forward 5′-CTAGGCACCAGGGTGTGATG-3′ and reward 5′-CTACGTACATGGCTGGGGTG-3′ for mouse *β*-actin (PCR product size is 291 bp). The reaction mixture containing cDNA was preheated for 5 minutes at 95°C as an initial denaturation step. Polymerase chain reaction consisted of denaturation step for 20 seconds at 95°C, annealing step for 55°C at 10 seconds, extension step for 30 seconds at 72°C, and final extension step for 5 minutes at 72°C.

TRIzol reagent was purchased from Invitrogen (Carlsbad, California, USA). Maxime PCR Premix kit, cDNA synthesis kit, and PCR Premix were obtained from iNtRON Biotechnology (Gyeonggi-do, Korea).

### 2.8. Estimation of Succinate Dehydrogenase (SDH) Activity in the Liver

Tissue samples were homogenized in phosphate-buffered saline (PBS) containing 1% protease inhibitor. The homogenized extracts were centrifuged at 13,000 rpm, 4°C for 5 min. The supernatants were transferred into new tubes and mixed with incubation solution containing 1 mol/L phosphate buffer (25 *μ*L), 0.2 mol/L sodium succinate (125 *μ*L), 10 mg/mL nitroblue tetrazolium (NBT; 25 *μ*L), and distilled water (235 *μ*L). The mixture was incubated for 20 min at 37°C in a temperature-controlled chamber. Sodium succinate, nitroblue tetrazolium, and protease inhibitor were purchased from Sigma-Aldrich. An enzyme solution (90 *μ*L) was added to prewarmed incubation solution (410 *μ*L) and incubated at 37°C. After the reaction is complete, the absorbance of the reaction mixture was measured at a wavelength of 550 nm. Enzyme activity was calculated using the following formula: enzyme activity = absorbance of enzyme reaction mixture − absorbance of diluted enzyme solution.

### 2.9. Immunohistochemistry

Tissue slides were soaked in xylene to remove paraffin and then sequentially soaked in 100~75% ethanol solutions for dehydration. Deparaffinized and dehydrated slides were reacted with 3% H_2_O_2_ solution for 10 min and washed. The slides were then blocked with normal serum solution for 1 h. The slides were then treated with primary antibodies for 1 h and washed with Tris-buffered saline containing 0.05% Tween 20 (TBS-T). Primary antibodies for superoxide dismutase (SOD) were obtained from Novus (Littleton, CO, USA) and for HMGCR from Santa Cruz Biotechnology, Inc., and TNF*α* and 4-hydroxynonenal (4-HNE) antibodies from Abcam. Secondary antibodies were reacted to the slides for 30 min. After washing with TBS-T, premixed Vectastain® ABC reagent was reacted with the slides for 30 min. The slides were then washed with TBS-T and reacted with 3,3′-diaminobenzidine substrate solution until a change in color was observed. After washing with tap water for 5 min, the slides were counterstained with hematoxylin. The slides were again washed with tap water, air-dried, and finally mounted. Immunohistochemistry kit (containing secondary antibody) was purchased from Vector Laboratories (Burlingame, CA, USA).

### 2.10. Oil Red O Staining

Cells in 24-well culture plates were fixed in 4% formaldehyde solution for 30 min and then washed with PBS for 5 min. The cells were stained with Oil Red O solution (Sigma-Aldrich) for 1 h. After a 40% isopropyl alcohol wash for 30 s, cells were washed twice with PBS for 5 min. The cells were observed with an optical microscope and photographed. Absolute isopropyl alcohol (1 mL) was added to each well, and the eluted Oil Red O was quantified with Spectramax plus 384 microplate reader (Molecular Devices LLC, Sunnyvale, CA, USA) at a wavelength of 530 nm.

### 2.11. Western Blot Analysis

Protein concentration was estimated by the Bradford method. Extracted proteins (10 *μ*g) were loaded onto 10% sodium dodecyl sulfate (SDS) polyacrylamide gels and protein blotting on nitrocellulose membranes was performed for 90 min. The membranes were blocked overnight with 5% skim milk and washed three times for 10 min with TBS-T. Primary antibodies were bound to the membranes at room temperature for 2 h. Primary antibodies for total and phosphorylated forms of AMPK, ACC, and ATP citrate lyase (ACLY) were supplied by Cell Signaling Technology, Inc. (Danvers, MA, USA). Primary antibodies for peroxisome proliferator-activated receptor gamma coactivator 1-alpha (PGC-1*α*), PPAR*δ*, PPAR*α*, and FASN were purchased from Abcam. Primary antibody for *β*-actin was procured from Santa Cruz Biotechnology, Inc. Dilution conditions for primary antibodies were as follows: PPAR*δ* was 1 : 500, AMPK, p-AMPK (at Thr172), ACC, p-ACC (at Ser79), ACLY, p-ACLY (at Ser455), FASN, PPAR*α*, and PGC-1*α* were 1 : 1000, and *β*-actin was 1 : 800. After washing three times with TBS-T for 10 min, secondary antibodies (Santa Cruz Biotechnology, Inc.) were bound to the membranes at room temperature for 1 h. Dilution conditions for secondary antibodies were as follows: anti-rabbit IgG antibodies for PPAR*δ*, PPAR*α*, AMPK, p-AMPK, ACC, p-ACC, ACLY, p-ACLY, FASN, and PGC-1*α* were 1 : 5000, and anti-mouse IgG antibody for *β*-actin was 1 : 5000. After three washes with TBS-T for 10 min and a single TBS wash for another 10 min, chemiluminescent substrate and enhancer solution (Bio-Rad, Hercules, CA, USA) were applied to membranes to determine the protein expression rate. Images were processed manually with Kodak GBX developer and fixer reagents (Carestream Health, Inc., Rochester, NY, USA) and analyzed using Image J program. *β*-Actin was used as the internal control to normalize the loaded proteins.

### 2.12. Hematoxylin and Eosin (H&E) Staining

The liver tissue of each mouse was harvested and fixed in 4% paraformaldehyde. The samples were embedded in paraffin, cut into 4~5 *μ*m thick sections using a microtome, and stained with H&E (Sigma-Aldrich). The sections were visualized using an optical microscope (BX51; Olympus, Tokyo, Japan) and photographed.

### 2.13. Immunocytochemistry

Cells in chamber slide were fixed with ice-cold methanol for 15 min. The intrinsic peroxidase activity in cells was get rid of by treatment of PBS containing 0.3% H_2_O_2_ and 0.3% normal serum. After PBS washing for 5 min, PBS containing 0.25% Triton X-100 was added to cells and incubated for 10 min. After PBS washing for 5 min, the cells were incubated for 20 min with normal blocking serum which was diluted in PBS as ratio of 1 : 100. After removing blocking serum, the cells were incubated for 1 hr with primary antibody solution (PPAR*γ* primary antibody was purchased from Abcam). After PBS washing for 5 min, secondary antibody solution was added to cells and incubated 30 min. After PBS washing for 5 min, Vectastain® ABC reagent was added to the cells and incubated for 30 min. After PBS washing for 5 min, 3,3′-diaminobenzidine (DAB) substrate solution was added and incubated until proper color change appeared. After washing three times with PBS, the cells were counterstained with hematoxylin. After washing with distilled water, the cells in chamber slide were dried, covered with glass, and then observed with optical microscope. Immunohistochemistry kit (containing secondary antibody) was purchased from Vector Laboratories.

### 2.14. Statistics

Data are presented as mean ± SEM (standard error of mean). Statistically significant differences between two groups were calculated by the unpaired *t*-test, and one way ANOVA test was used to compare means of three or more groups. The *p* value of <0.05 was considered significant.

## 3. Results

### 3.1. HPLC Analysis Profile of Ethyl Acetate Extract of* A. glehni* Showed That the Extract Contains Mainly Caffeoylquinic Acids

**Figure 1:**
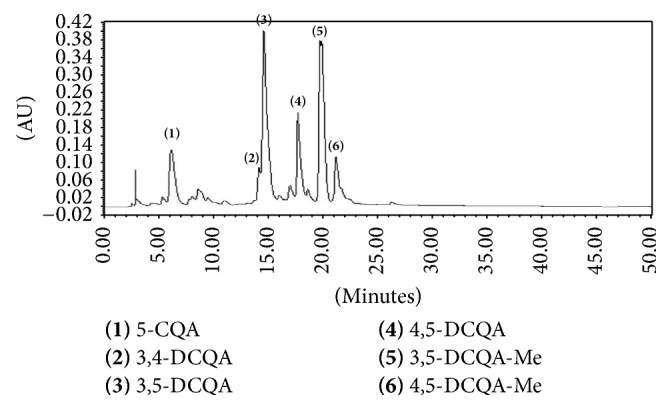
HPLC profile for ethyl acetate extract of* Aster glehni.* Separated phytochemicals are as follows: (1) 5-CQA: 5-caffeoylquinic acid, (2) 3,4-DCQA: 3,4-dicaffeoylquinic acid, (3) 3,5-DCQA: 3,5-dicaffeoylquinic acid, (4) 4,5-DCQA: 4,5-dicaffeoylquinic acid, (5) 3,5-DCQA-Me: methyl 3,4-dicaffeoylquinic acid, and (6) 4,5-DCQA-Me: methyl 4,5-dicaffeoylquinic acid.

Ethyl acetate extract of* A. glehni *contains mainly caffeoylquinic acids of six kinds, and they are as follows: 5-caffeoylquinic acid, 3,4-dicaffeoylquinic acid, 3,5-dicaffeoylquinic acid, 4,5-dicaffeoylquinic acid, methyl 3,4-dicaffeoylquinic acid, and methyl 4,5-dicaffeoylquinic acid (Figure 1).

### 3.2. Weight of Body and Abdominal (Epididymal) Fat and Concentrations of Triglyceride, Total Cholesterol, HDL Cholesterol, LDL Cholesterol, and Adiponectin in the Serum of ApoE KO Mice Treated with 0.15% Cholesterol Diet and* A. glehni* Extracts Were Normalized by the Extracts

**Figure 2:**
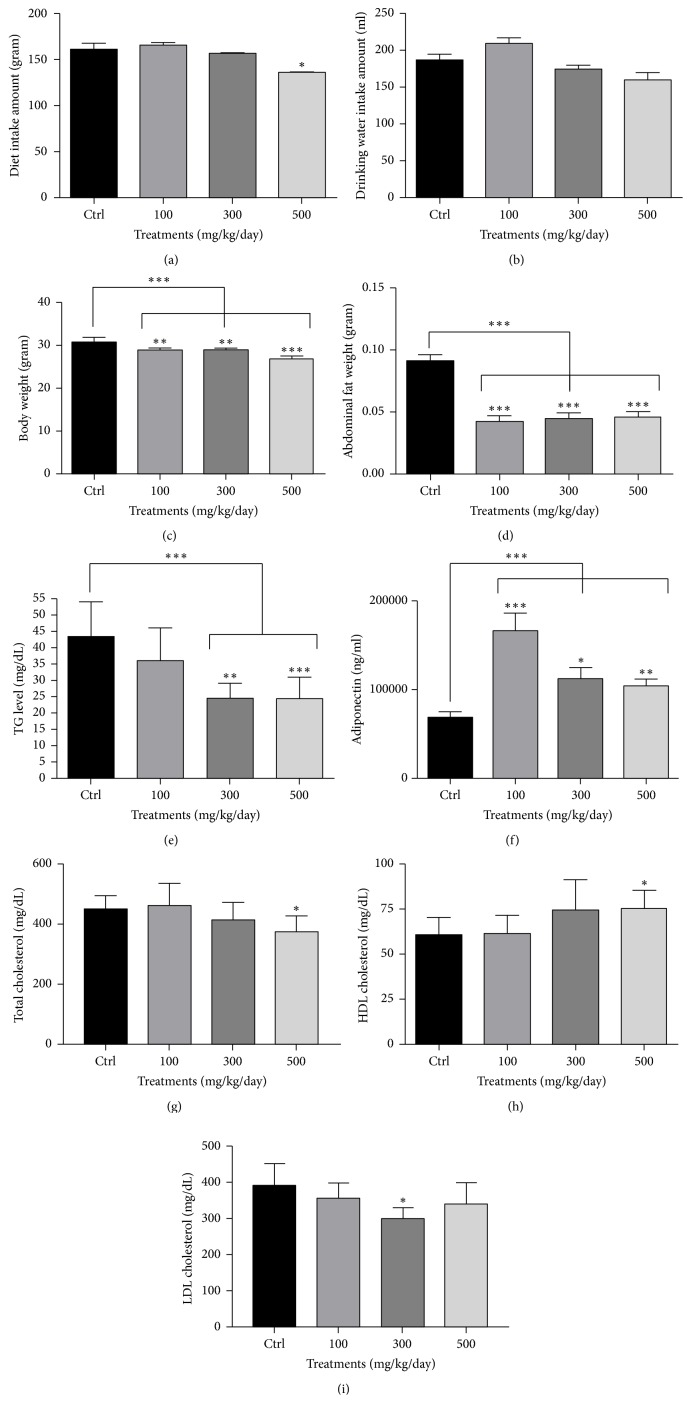
Effects of* Aster glehni *extract on diet intake amount, drinking water intake amount, body weight, abdominal adipose tissue weight, serum triglyceride, and serum adiponectin, total cholesterol, high-density lipoprotein (HDL) cholesterol, low-density lipoprotein (LDL) cholesterol in ApoE KO mice administered with 0.15% cholesterol diet. ((a) and (b)) Diet and drinking water intake amounts were estimated for four weeks. (c) Body weight of mice was estimated just prior to sacrifice. (d) Abdominal adipose tissue was removed from sacrificed mice and weighed. (e) Triglyceride concentration in serum was estimated with triglyceride colorimetric assay kit. (f) Adiponectin concentration in serum was estimated with mouse adiponectin enzyme-linked immunosorbent assay (ELISA) kit. (g, h, and i) Levels of cholesterols were estimated with triglyceride colorimetric assay kit. The results are expressed as means ± SEM. Values were statistically analyzed by unpaired *t*-test and one way ANOVA. All experiments were repeated three times. ^*∗*^*p* < 0.05 versus control, ^*∗∗*^*p* < 0.01 versus control, and ^*∗∗∗*^*p* < 0.001 versus control.

The diet intake amount was not significantly different among experimental groups except for* A. glehni *extract treated group of 500 mg/kg/day (Figure 2(a)). Likewise, the drinking water intake amount was not different among all experimental groups (Figure 2(b)).

Body and abdominal (epididymal) adipose tissue weights of mice treated with* A. glehni* extract were significantly lower than those of control mice (Figures 2(c) and 2(d)). So the correlation between diet intake amount and body weight was not obvious.

The concentration of triglyceride in serum decreased in a concentration-dependent manner and significant differences were observed in groups treated with 300 and 500 mg/kg/day* A. glehni* extract (Figure 2(e)). Adiponectin is a glycosylated adipokine that is selectively secreted by adipocytes. It has been reported to improve insulin sensitization, stimulate fatty acid oxidation, reduce inflammation, and inhibit atherosclerosis [15]. Adiponectin level increased upon* A. glehni* extract treatment at all concentrations (Figure 2(f)). On the other hand, cholesterol levels changed according to the concentration of* A. glehni* administered: total cholesterol level decreased significantly after treatment with 500 mg/kg/day of the extract (Figure 2(g)), HDL cholesterol level increased after the same treatment (Figure 2(h)), and LDL cholesterol level decreased after treatment with 300 mg/kg/day of the extract (Figure 2(i)).

### 3.3. Semiquantitative RT-PCR Results for PPAR*δ*, AMPK*α*1, IL6, and TNF*α* in the Liver of ApoE KO Mice Treated with 0.15% Cholesterol Diet and* A. glehni* Extracts Showed the Elevation of PPAR*δ* and AMPK*α*1 and the Decrease of IL6 and TNF*α*

**Figure 3:**
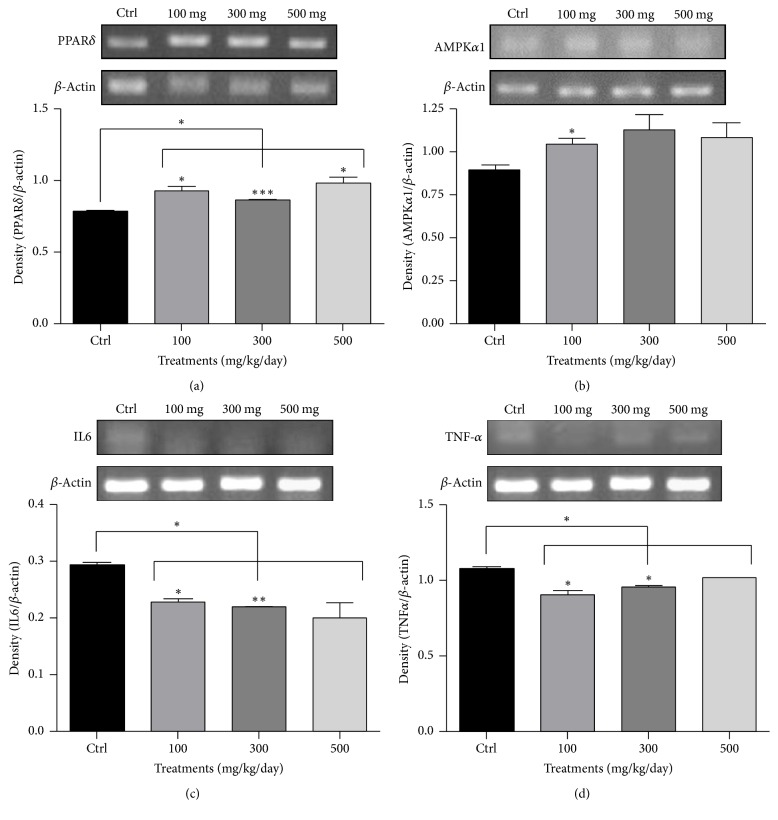
The mRNA levels of peroxisome proliferator-activated receptor delta (PPAR*δ*), 5′ adenosine monophosphate-activated protein kinase *α*1 (AMPK*α*1), interleukin 6 (IL6), and tumor necrosis factor *α* (TNF*α*) in liver of ApoE KO mice treated with 0.15% cholesterol diet and* Aster glehni *extract. Total RNA was extracted by Trisol reagent from liver tissues of ApoE KO mice, and complementary DNA was synthesized by cDNA synthesis kit from the total RNA. Polymerase chain reaction (PCR) was done with cDNA, primers, and PCR Premix solution. The PCR image density was analyzed with Image J program. The results are expressed as means ± SEM. Values were statistically analyzed by unpaired *t*-test and one way ANOVA. All experiments were repeated three times. ^*∗*^*p* < 0.05 versus control, ^*∗∗*^*p* < 0.01 versus control, and ^*∗∗∗*^*p* < 0.001 versus control.

The mRNA level for PPAR*δ* was increased by* A. glehni* extracts of all concentrations (Figure 3(a)), and the expression of AMPK*α*1 catalytic subunit was significantly elevated with the extract treatment of 100 mg/kg/day concentration (Figure 3(b)). The mRNA levels for IL6 and TNF*α* which are involved in inflammation were lowered by the extracts of all concentrations (Figures 3(c) and 3(d)).

### 3.4. Western Blot Analyses of PPAR*δ*, AMPK, ACC, ACLY, PGC-1*α*, and FASN Expression in the Liver of ApoE KO Mice Treated with 0.15% Cholesterol Diet and* A. glehni* Extracts Showed the Activation of Fatty Acid Oxidation and the Inhibition of Fatty Acid Synthesis by the Extracts

Phosphorylated AMPK (P-AMPK) is the active form of the enzyme and regulates the activation of ACC. Once ACC is phosphorylated, it becomes inactivated and no longer catalyzes the production of malonyl-CoA—an essential component in fatty acid synthesis [16]. The function of ACLY is to link glucose and lipid metabolisms. In its active form, phosphorylated ACLY transforms citrate into acetyl-CoA, which can be used as a substrate in the mevalonate and fatty acid synthesis pathways [17, 18]. The transcriptional coactivator PGC-1*α* coordinately increases mitochondrial biogenesis and respiration rates, as well as the uptake and utilization of substrates for energy production [19].

**Figure 4:**
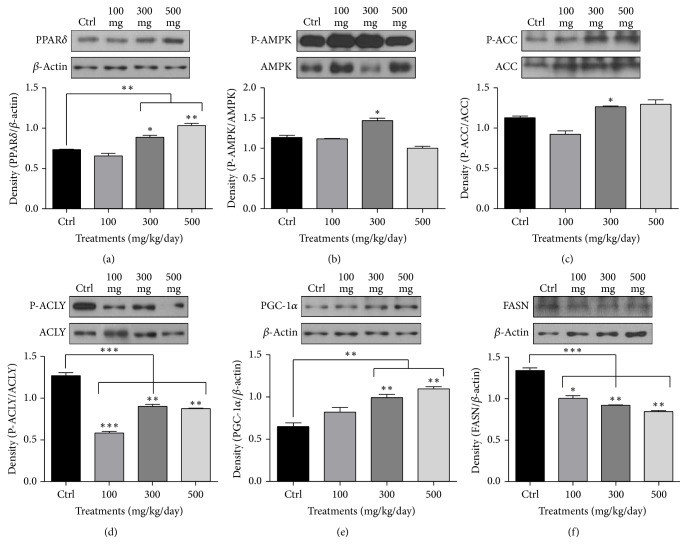
The protein levels of peroxisome proliferator-activated receptor delta (PPAR*δ*), 5′ adenosine monophosphate-activated protein kinase (AMPK), acetyl-CoA carboxylase (ACC), ATP citrate lyase (ACLY), peroxisome proliferator-activated receptor gamma coactivator 1-alpha (PGC-1*α*), and fatty acid synthase (FASN) in liver of ApoE KO mice treated with 0.15% cholesterol diet and* Aster glehni *extract. Protein extracts were electrophoresed in 10% polyacrylamide gel and blotted to nitrocellulose membrane. The nitrocellulose membrane was bound with primary and secondary antibodies sequentially, and then the chemiluminescence was exposed to X-ray film. The density for bands on X-ray film was analyzed with Image J program. The results are expressed as means ± SEM. Values were statistically analyzed by unpaired *t*-test and one way ANOVA. All experiments were repeated three times. ^*∗*^*p* < 0.05 versus control, ^*∗∗*^*p* < 0.01 versus control, and ^*∗∗∗*^*p* < 0.001 versus control.

The protein level of PPAR*δ* increased significantly in the liver of experimental groups treated with 300 and 500 mg/kg/day* A. glehni* extracts (Figure 4(a)). Similarly, the expression of P-AMPK increased significantly in the experimental group treated with 300 mg/kg/day* A. glehni* extract (Figure 4(b)). The expression of phosphorylated ACC (P-ACC) was significantly elevated in the groups treated with 300 mg/kg/day* A. glehni* extract (Figure 4(c)). Conversely, the expression of phosphorylated ACLY (P-ACLY) decreased significantly in all the groups given 100, 300, and 500 mg/kg/day* A. glehni* extracts (Figure 4(d)). Protein level of PGC-1*α* increased in groups treated with 300 and 500 mg/kg/day* A. glehni* extracts (Figure 4(e)), whereas that of FASN decreased in all groups treated with* A. glehni* extracts (Figure 4(f)).

### 3.5. Immunohistochemistry Analyses of TNF*α*, SOD, HMGCR, and 4-HNE Expression in Liver Sections of ApoE KO Mice Treated with 0.15% Cholesterol Diet and* A. glehni* Extracts Showed the Decreases of TNF*α* and HMGCR and the Increase of SOD

It is generally known that hyperlipidemia aggravates inflammation and stimulates the generation of reactive oxygen species [20, 21]. The level of inflammation in the body can be estimated by the level of inflammatory cytokines such as TNF*α*; in this study, the level of TNF*α* decreased in all groups treated with* A. glehni *extracts. On the contrary, the expression of SOD, an enzyme that converts O_2_^−^ into either molecular oxygen (O_2_) or hydrogen peroxide (H_2_O_2_), increased in all groups treated with* A. glehni *extracts. The expression of HMGCR in the control group was clearly evident; however, its expression was attenuated in experimental groups treated with* A. glehni *extracts.

**Figure 5:**
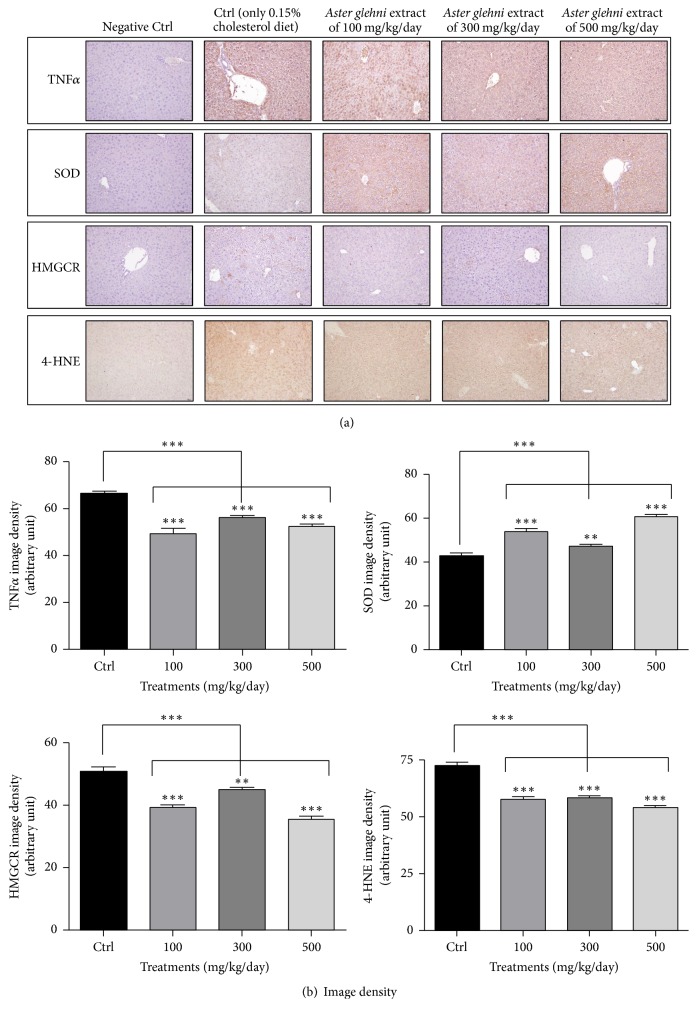
Immunohistochemistry for TNF*α*, SOD, HMGCR, and 4-HNE on frozen sectioned slides of liver in ApoE KO mice treated with 0.15% cholesterol diet and* Aster glehni *extract. (a) Liver tissue slides of ApoE KO mice were fixed and immunohistochemically stained with antibodies of TNF*α*, SOD, HMGCR, and 4-HNE. Images were taken at 200x magnification. (b) Densities for images were analyzed with Image J program. The results are expressed as means ± SEM. Values were statistically analyzed by unpaired *t*-test and one way ANOVA. All experiments were repeated three times. ^*∗∗*^*p* < 0.01 versus control and ^*∗∗∗*^*p* < 0.001 versus control.

4-Hydroxynonenal (4-HNE) is generally known with a product of lipid peroxidation and an inducer of oxidative stress, and it was reported to accelerate the lipid accumulation in hepatocytes [22]. In addition, 4-HNE protein expression was decreased by AMPK agonist, 5-aminoimidazole-4-carboxamide-1-beta-D-ribofuranoside (AICAR) in alcohol induced fatty liver [23]. The 4-HNE expression was decreased with* A. glehni* extracts (Figure 5).

### 3.6. In the Liver of ApoE KO Mice Treated with 0.15% Cholesterol Diet and* A. glehni* Extracts, Succinate Dehydrogenase Activity Was Increased, Concentrations of Triglyceride, IL6, and ROS Were Decreased, and the Morphology of Hepatocytes Was Improved in the Group Treated with the Extract of 300 mg/kg/day

**Figure 6:**
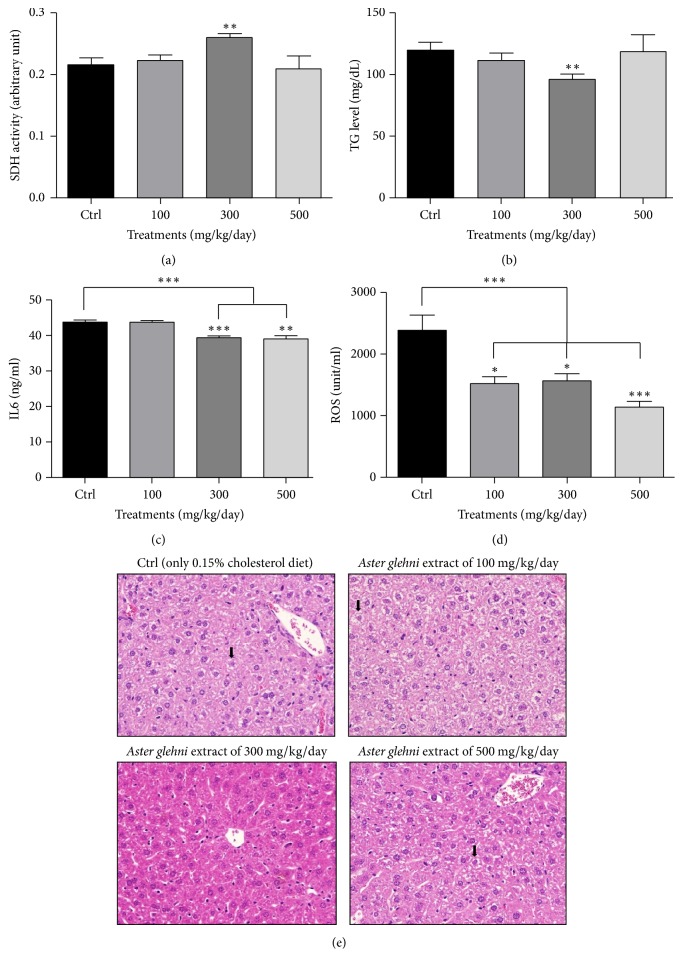
Succinate dehydrogenase activity, triglyceride, interleukin 6 (IL6), and reactive oxygen species (ROS) concentrations, and hematoxylin and eosin staining in liver of ApoE KO mice treated with 0.15% cholesterol diet and* Aster glehni *extract. (a) Succinate dehydrogenase (SDH) activity in liver protein extracts of ApoE KO mice was estimated with colorimetric method containing nitroblue tetrazolium reagents. (b) Triglyceride (TG) contents in liver extracts of ApoE KO mice were estimated with triglyceride colorimetric assay kit. (c, d) IL6 and ROS contents in liver extracts of ApoE KO mice were estimated with IL6 and ROS colorimetric assay kits. (e) Liver tissue slides of ApoE KO mice were fixed and stained by hematoxylin and eosin reagents. Magnification is 200 times. The results are expressed as means ± SEM. Values were statistically analyzed by unpaired *t*-test and one way ANOVA. All experiments were repeated three times. Liver cells having representative ballooning morphology were indicated by arrow marks (↓). ^*∗*^*p* < 0.05 versus control, ^*∗∗*^*p* < 0.01 versus control, and ^*∗∗∗*^*p* < 0.001 versus control.

Succinate dehydrogenase catalyzes the reaction that converts succinate and flavin adenine dinucleotide (FAD) into fumarate and FADH2 in the citric acid cycle, also it participates in the electron transport chain known. In addition, the SDH is known as a biomarker of mitochondrial biogenesis, together with PGC-1*α* [24]. In this study, SDH activity increased only in mice treated with 300 mg/kg/day* A. glehni* (Figure 6(a)). Triglyceride concentration in the liver decreased significantly in the experimental group treated with 300 mg/kg/day* A. glehni* (Figure 6(b)). Ballooning of hepatocytes is an important index for the histological diagnosis of NASH [25]. IL6 concentration in the liver decreased in the experimental groups treated with* A. glehni* extracts of 300 and 500 mg/kg/day concentrations (Figure 6(c)). Also the concentration of reactive oxygen species (ROS) was decreased in experimental groups treated with* A. glehni* extracts of all concentrations (Figure 6(d)).

Balloon-like cell morphology was observed in all experimental groups except for the group treated with 300 mg/kg/day* A. glehni*, which exhibited liver morphology that was most similar to that of a normal liver (Figure 6(e)). These results suggested that a 300 mg/kg/day dosage of* A. glehni* was most effective in preventing and ameliorating fatty liver in ApoE KO mice given 0.15% cholesterol diet.

### 3.7. Oil Red O Staining and Western Blot Analyses for PPAR*δ*, AMPK, and PGC-1*α* in HepG2 Cells Treated with* A. glehni* Extract and GSK0660 in High Fatty Acid and High Cholesterol Condition Showed the Direct Decrease of Lipid Accumulation and the Increases of Biomarkers Related to Lipid Oxidation

**Figure 7:**
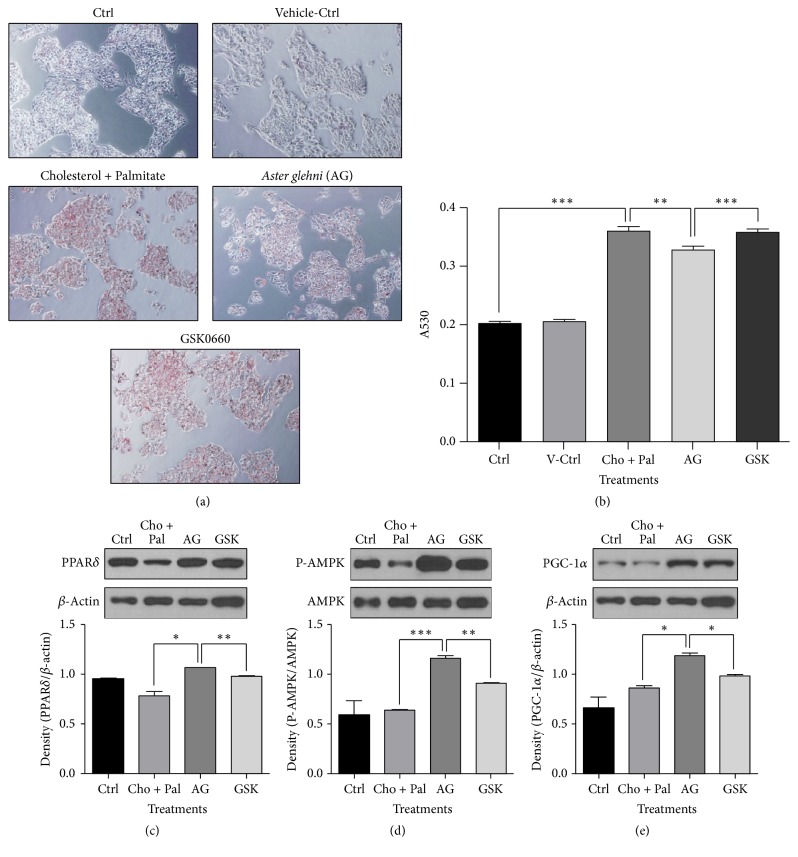
Oil Red O staining result and the protein levels of PPAR*δ*, AMPK and PGC-1*α* in HepG2 cells treated with 0.2 mM cholesterol, 0.1 mM palmitate, 50 ug* Aster glehni *extract, and 50 uM GSK0660 (PPAR*δ* antagonist). The lipids in HepG2 (a) were stained with Oil Red O reagent and observed by optical microscope. Accumulated lipid contents in HepG2 cells (b) were eluted by isopropanol and the absorbance of eluted Oil Red O was estimated with ELISA reader at the wave length of 530 nm. Magnification is 200 times. Meaning of indications: Ctrl is an untreated control group, V-Ctrl is a vehicle control group, Cho + Pal is a cholesterol and palmitate treated group, AG is a cholesterol, palmitate, and* A. glehni *treated group, and GSK is a cholesterol, palmitate,* A. glehni*, and GSK0660 treated group. Protein extracts were electrophoresed in 10% polyacrylamide gel and blotted to nitrocellulose membrane. The nitrocellulose membrane was bound with primary and secondary antibodies sequentially, and then the chemiluminescence was exposed to X-ray film. The density for bands on X-ray film was analyzed with Image J program (c, d, e). The results are expressed as means ± SEM. Values were statistically analyzed by unpaired *t*-test and one way ANOVA. All experiments were repeated three times. ^*∗*^*p* < 0.05, ^*∗∗*^*p* < 0.01, and ^*∗∗∗*^*p* < 0.001.

While the lipid content in HepG2 cells increased to 77.6% upon the cotreatment of palmitate and cholesterol compared to control, the elevated lipid level decreased significantly in cotreating condition of palmitate, cholesterol, and* A. glehni* extract. But the effect of* A. glehni* was inhibited by the cotreatment of PPAR*δ* antagonist, GSK0660 (Figures 7(a) and 7(b)). Protein levels of PPAR*δ*, P-AMPK, and PGC-1*α* increased upon* A. glehni* extract treatment compared with their levels in the control group. However, this effect of* A. glehni* extract was suppressed by the action of the PPAR*δ* antagonist, GSK0660 (Figures 7(c)–7(e)).

### 3.8. Protein Expression Levels for PPAR*γ* and PPAR*α* Were Not Changed with* A. glehni* Extract in HepG2 Cells in High Fatty Acid and High Cholesterol Condition: AMPK Antagonist, Compound C, Did Not Affect the Protein Level of PPAR*δ*

**Figure 8:**
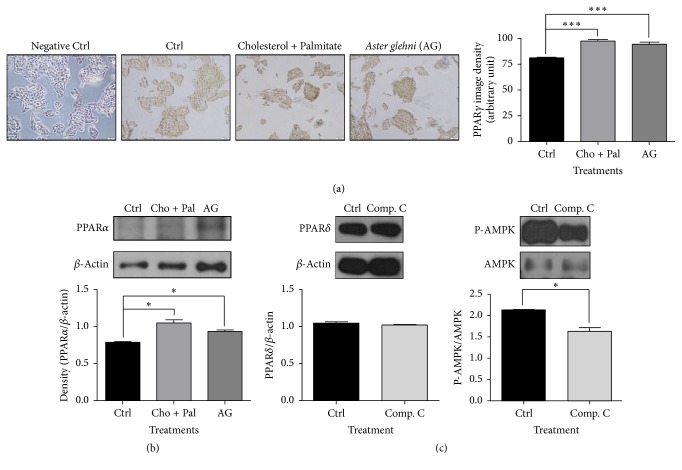
The protein levels of PPAR*γ* and PPAR*α* in HepG2 cells treated with 0.2 mM cholesterol, 0.1 mM palmitate, and 50 ug* Aster glehni *extract, and PPAR*δ* and AMPK protein levels in HepG2 cells treated with 10 uM compound C (AMPK antagonist). (a) Liver tissue slides of ApoE KO mice were fixed and immunohistochemically stained with antibody of PPAR*γ*. Images were taken at 200x magnification. (b, c) Protein extracts were electrophoresed in 10% polyacrylamide gel and blotted to nitrocellulose membrane. The nitrocellulose membrane was bound with primary and secondary antibodies sequentially, and then the chemiluminescence was exposed to X-ray film. The density for bands on X-ray film was analyzed with Image J program. The results are expressed as means ± SEM. Values were statistically analyzed by unpaired *t*-test and one way ANOVA. All experiments were repeated three times. ^*∗*^*p* < 0.05 and ^*∗∗∗*^*p* < 0.001.

*Aster glehni* extract did not change the protein expression levels for PPAR*γ* and PPAR*α* in HepG2 cells treated with high fatty acid and high cholesterol (Figures 8(a) and 8(b)). The AMPK antagonist (compound C) did not reduce the PPAR*δ* protein level (Figure 8(c)).

### 3.9. Oil Red O Staining of Differentiated 3T3-L1 Cells Treated with* A. glehni* Extract and GSK0660 in High Fatty Acid and High Cholesterol Condition Showed the Antiadipogenic Effect of the Extract, and Protein Levels for PPAR*δ*, AMPK, and PGC-1*α* in Abdominal Fat Tissues from ApoE KO Mice Given 0.15% Cholesterol Diet Were Mainly Increased in the Group Treated with the Extract of 500 mg/kg/day

**Figure 9:**
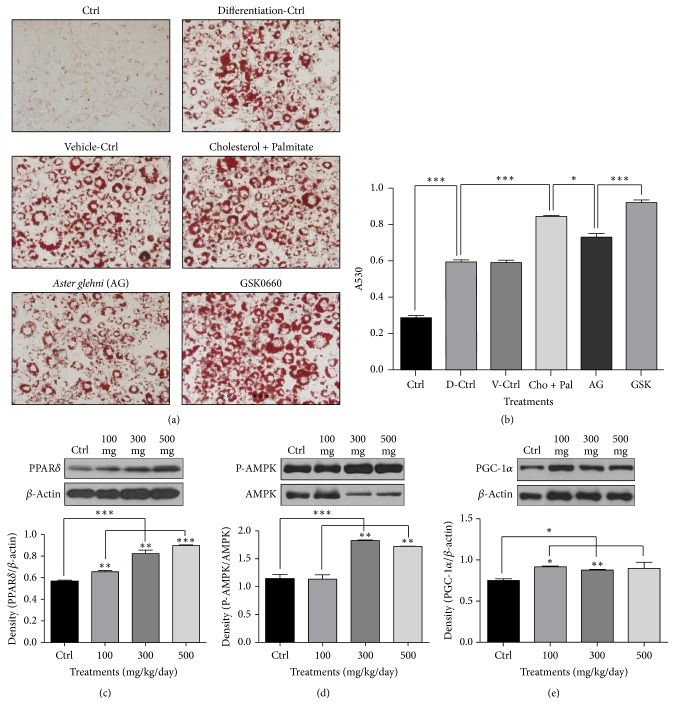
Oil Red O staining results in differentiated 3T3-L1 cells treated with 0.2 mM cholesterol, 0.1 mM palmitate, 50 ug* Aster glehni *extract, and 50 uM GSK0660 (PPAR*δ* antagonist) and the protein levels of PPAR*δ*, AMPK, and PGC-1*α* in abdominal fat tissues of ApoE KO mice treated with 0.15% cholesterol diet and* Aster glehni *extract. The lipids in differentiated 3T3-L1 cells (a) were stained with Oil Red O reagent and observed by optical microscope. Accumulated lipid contents in differentiated 3T3-L1 cells (b) were eluted by isopropanol and the absorbance of eluted Oil Red O was estimated with ELISA reader at the wave length of 530 nm. Magnification is 200 times. Meaning of indications: Ctrl is an untreated control group, D-Ctrl is a differentiation control group, V-Ctrl is a vehicle control group, Cho + Pal is a cholesterol and palmitate treated group, AG is a cholesterol, palmitate, and* A. glehni* treated group, and GSK is a cholesterol, palmitate,* A. glehni*, and GSK0660 treated group. Protein extracts were electrophoresed in 10% polyacrylamide gel and blotted to nitrocellulose membrane. The nitrocellulose membrane was bound with primary and secondary antibodies sequentially, and then the chemiluminescence was exposed to X-ray film. The density for bands on X-ray film was analyzed with Image J program (c, d, e). The results are expressed as means ± SEM. Values were statistically analyzed by unpaired *t*-test and one way ANOVA. All experiments were repeated three times. ^*∗*^*p* < 0.05, ^*∗∗*^*p* < 0.01, and ^*∗∗∗*^*p* < 0.001.

The lipid content in differentiated 3T3-L1 cells, which was increased by palmitate and cholesterol treatment, decreased upon treatment with* A. glehni*, and the effect of* A. glehni* on these cells was also suppressed by the cotreatment of GSK0660 (Figures 9(a) and 9(b)). Protein levels for PPAR*δ*, P-AMPK, and PGC-1*α* were highest in abdominal fat from mice treated with 500 mg/kg/day* A. glehni* compared to control (Figures 9(c)–9(e)).

## 4. Discussion

The liver has many essential physiological functions such as hematopoiesis, secretion of bile, eradication of foreign material and bacteria by Kupffer cells, and maintaining energy homeostasis of the body [26]. Many studies have reported that abnormal lipid metabolism in the liver is related to various metabolic diseases such as atherosclerosis, obesity, type 2 diabetes, and hypertension. For instance, it has been reported that the risk of hypertension and type II diabetes is higher in patients with NAFLD than in those in the non-NAFLD group [27–30]. Moreover, carotid intima-media thickness is greater in patients with NAFLD than in the normal healthy population [31]. In obese persons, excess intrahepatic triglyceride is a strong indicator of metabolic abnormalities and is independent from other metabolic measurements like body mass index, percent body fat, and visceral fat mass [32]. Hence, metabolic functions of the liver are vital to maintain metabolic homeostasis and the overall health of the body.

**Figure 10:**
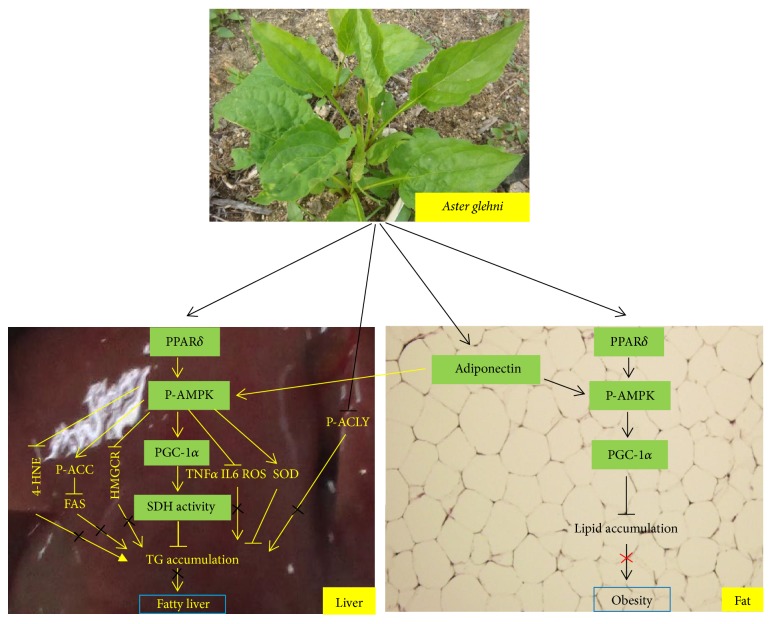
Schematic diagram for the functional mechanism of* Aster glehni *extract. Amelioration of nonalcoholic fatty liver by* Aster glehni* is mainly accomplished with catabolic activation such as the sequential regulation of PPAR*δ*/adiponectin→P-AMPK→PGC-1*α*→SDH, also it is done by other pathway of P-AMPK→P-ACC which inhibits fatty acid synthesis.* Aster glehni *additionally lowers triglyceride accumulation in liver by inhibiting P-ACLY, also its anti-TG accumulation effect is done through the regulation of TNF*α* and SOD by P-AMPK. In addition, the extract may prevent obesity via PPAR*δ*/adiponectin→P-AMPK→PGC pathway. Meaning of symbols: arrow means activation, up and horizontal line means inhibition, and × means blocking. Photograph of* Aster glehni* was quoted from Korea Rural Development Administration Genebank information center (PCV0039).

In our study for the effect and the mechanism of* Aster glehni* on NAFLD in ApoE KO mice, the concentration of adiponectin in serum was significantly higher in the experimental groups treated with* A. glehni* extracts than that of the control group. Moreover, the concentration of triglyceride in serum, the weight of abdominal adipose tissue, and the weight of body were lower in the* A. glehni* treated groups than in the untreated group. And* A. glehni* extract sequentially regulated genes involved in fatty acid metabolism. For instance, upregulation of adiponectin and PPAR*δ* by* A. glehni* induced AMPK activation, which consequently increased the levels of PGC-1*α* and p-ACC; this in turn elevated SDH activity and decreased FASN expression. In addition,* A. glehni* suppressed protein levels of p-ACLY and HMGCR. Hence, the final outcome of* A. glehni* action is the decrease of triglyceride level in the liver (Figures 6 and 10). From the data for TG level and SDH (Figure 6), the most effective treatment concentration of* Aster glehni* extract to prevent NAFLD in atherosclerotic condition is supposed to be 300 mg/kg/day. But the* A. glehni* extract showed minus effects at the concentration over 300 mg/kg/day in liver lipid metabolism, and it is supported by the following result: the TG level in liver showed an elevated tendency in the group treated with extract of 500 mg/kg/day concentration compared to group treated with the extract of 300 mg/kg/day concentration. So, it can be postulated that the* A. glehni* extract of high concentration has negative effects in the liver lipid metabolism of ApoE KO mice.

*A. glehni* treatment may additionally ameliorate NAFLD via regulation of SOD, TNF*α*, 4-HNE, IL6, and ROS (Figures 5, 6, and 10). In this study, the cause for the reduction of body weight and abdominal adipose tissue mass by the treatment of* A. glehni* extract can be explained from the elevation of biomarkers related to fatty acid oxidative metabolism and the downregulation of biomarkers involved in lipid synthesis and inflammation. The PPAR*δ* antagonist (GSK0660) offset the effects of* A. glehni* stimulating protein expression of PPAR*δ*, p-AMPK, and PGC-1*α* in HepG2 cells (Figure 7) and reversed its effect of lowering lipid accumulation in HepG2 and differentiated 3T3-L1 cells (Figures 7 and 9). These results indicate that PPAR*δ* regulates AMPK and PGC-1*α*. In addition, the* A. glehni* extract did not affect protein expression for PPAR*α* and PPAR*γ* in the condition of cholesterol and palmitate cotreatment (Figures 8(a) and 8(b)), so it can be supposed that* A. glehni* extract specifically regulates PPAR*δ* compared to PPAR*α* and PPAR*γ*. In addition, PPAR*δ* antagonist (GSK0660) decreased the protein level of P-AMPK (Figure 7(d)), but the AMPK antagonist (compound C) did not change the PPAR*δ* protein level (Figure 8(c)). So these results mean that PPAR*δ* is an upper regulator against AMPK.

Our results are almost consistent with the results of other papers as follows.

In adipose tissues, inflammatory proteins such as C-reactive protein and TNF*α* are negatively regulated by adiponectin; adiponectin also suppresses TNF*α* production in cardiac cells [33]. Furthermore, it specifically suppresses TNF*α*-induced activation of inhibitor of *κ*B- (I*κ*B-) *α*/nuclear factor *κ*B (NF-*κ*B) through the cAMP-dependent pathway in human aortic endothelial cells [34]. Adiponectin level in plasma of patients with metabolic diseases such as hypertension, type 2 diabetes, and obesity is lower than that of healthy subjects [35–37]. Adiponectin activates AMPK via phosphorylation. In turn, activated AMPK inactivates ACC by phosphorylation and decreases the production of malonyl-CoA, which is required in de novo fatty acid synthesis [16]. Also many studies in which PPAR*δ* regulates AMPK activation were reported as follows: the activated PPAR*δ* induced AMPK activation and affected glucose transport in skeletal muscle cells [38], PPAR*δ* activation by agonist induced the normalization of p-AMPK and PGC-1*α* levels lowered in liver of mice given high fat diet [39], and angiotensin II receptor blocker, telmisartan, decreased the weight gain and elevated the running endurance through the regulation of PPAR*δ*-AMPK pathway in which the activation of AMPK depends on PPAR*δ* [40]. The role of AMPK in regulating lipid metabolism has been described extensively. For example, intracellular triglyceride accumulation induced by TNF*α* decreased with administration of AMPK agonists such as metformin and 5-aminoimidazole-4-carboxamide-1-*β*-D-ribofuranoside (AICAR) in HepG2 cells. In addition, these AMPK agonists suppressed mechanistic target of rapamycin (mTOR) and p70S6K phosphorylation and reduced the levels of sterol regulatory element-binding protein-1 (SREBP-1) and FASN [41]. So it can be supposed that AMPK can lower the expression of inflammatory proteins such as TNF*α* as well as other proteins involved in fatty acid synthesis. Recently, Zhou et al. [42] demonstrated that the antiadipogenic effects of alpha-linolenic acid were dependent on AMPK and that its effects were abolished in AMPK*α*1 and AMPK*α*2 KO mice. In addition, the activation of AMPK by AICAR and metformin increases the activity of SOD. On the other hand, AMPK antagonist like compound C decreases the activity of SOD [43]. Moreover, resveratrol was shown to increase SOD level through an AMPK-dependent mechanism in vascular endothelial cells that were subjected to high glucose-induced oxidative stress conditions [43]. However, it was reported that constitutive activation of endothelial AMPK*α*1 promoted vascular inflammation and obesity-induced fatty liver, largely via induction of cyclooxygenase-2 [44]. In cholesterol metabolism, AMPK activation by AICAR inhibited the expression of SREBP-2 and its target genes, HMGCR and 3-hydroxy-3-methylglutaryl-CoA synthase (HMGCS), which are key enzymes in cholesterol biosynthesis [45]. Based on these studies on the relation between adiponectin/PPAR*δ* and lipid metabolism, it appears that adiponectin and PPAR*δ* regulate lipid metabolism through AMPK activation in the liver.

Adiponectin deficiency can induce NASH through decreased expression of genes related to mitochondrial biogenesis such as PGC-1*α*, regarded as the master regulator of mitochondrial biogenesis, in hepatocytes [46]. Both AMPK and NAD-dependent deacetylase sirtuin-1 (SIRT1) have been shown to directly affect PGC-1*α* activity [19]. Moreover, PGC-1*α* expression corresponds to severe metabolic changes as a result of exercise, starvation, and cold [47]. One study reported that PPAR*δ* activation by agonists upregulated PGC-1*α* mRNA expression via a PPAR-response element in the PGC-1*α* promoter [48]. Succinate dehydrogenase is involved in both citric acid cycle and electron transport chain and plays essential roles in oxidative metabolism together with PGC-1*α*. In obese patients with type 2 diabetes, rosiglitazone increased insulin sensitization through upregulation of mRNA levels of PPAR*δ* and PGC-1*α*, as well as stimulation of SDH activity in skeletal muscles [49]. In a metabolic syndrome rat model (SHR/NDmcr-cp) representing characteristics of hypertension and obesity, exercise normalized the activity of SDH and the mRNA expression of PPAR*δ* and PGC-1*α* [50]. Therefore, it can be supposed that the stimulation of PPAR*δ*, PGC-1*α*, and SDH induces the activation of catabolic metabolism causing energy expenditure, as well as a decrease in triglyceride and increase in adiponectin levels in serum. Furthermore, succinate treatment increased protein levels of *α*-smooth muscle actin and G protein-coupled receptor 91, which are essential markers of fibrogenesis in human hepatic stellate cells [51], suggesting the significance of SDH in improving liver diseases like cirrhosis.

Consequently, our results suggest that* A. glehni* extract improves abnormal metabolic profiles in serum and liver through accelerating energy expenditure and stimulating of lipid catabolism by PPAR*δ* and adiponectin and it can additionally ameliorate obesity through adiponectin/PPAR*δ*-AMPK-PGC-1*α* pathway (Figure 10). Because body and abdominal fat weights were significantly lowered at all treatment concentrations, the decrease of diet intake at high dose (500 mg/kg/day) may be due to the strong taste of* Aster glehni*; however, the exact mechanism will be explained by further study. In our research, the relation between PPAR*δ* and other biomarkers was relatively well studied; however, the reaction mechanism of adiponectin on lipid metabolism is deficient compared to PPAR*δ*. So in the following study, additional experiments for adiponectin are necessary.

Caffeoylquinic acid-rich* Pandanus tectorius* fruit extract ameliorates dyslipidemia and hyperglycemia via the activation of AMPK [52]. Also 5-caffeoylquinic acid improved obesity and fatty liver through the amelioration of lipid metabolism by PPAR*α* activation and LXR*α* inhibition [53]. In our study, the ethyl acetate extract of* A. glehni* contains mainly caffeoylquinic acids of six kinds. Therefore it can be supposed that the anti-NAFLD effect of* A. glehni* extract is dependent on caffeoylquinic acids. So in our following study, the anti-NAFLD effect and mechanism of caffeoylquinic acids purified from* A. glehni* should be additionally experimented.

## Figures and Tables

**Table 1:** Ingredients of cholesterol diet.

	Unit	Amount
*Macronutrients*		
Crude protein	%	18.6
Fat (ether extract)	%	6.2
Carbohydrate (available)	%	44.2
Crude fiber	%	3.5
Neutral detergent fiber	%	14.7
Ash	%	5.3
*Minerals*		
Calcium	%	1.0
Phosphorus	%	0.7
Non-phytate phosphorus	%	0.4
Sodium	%	0.2
Potassium	%	0.6
Chloride	%	0.4
Magnesium	%	0.2
Zinc	mg/kg	70
Manganese	mg/kg	100
Copper	mg/kg	15
Iodine	mg/kg	6
Iron	mg/kg	200
Selenium	mg/kg	0.23
*Amino acids*		
Aspartic acid	%	1.4
Glutamic acid	%	3.4
Alanine	%	1.1
Glycine	%	0.8
Threonine	%	0.7
Proline	%	1.6
Serine	%	1.1
Leucine	%	1.8
Isoleucine	%	0.8
Valine	%	0.9
Phenylalanine	%	1.0
Tyrosine	%	0.6
Methionine	%	0.4
Cystine	%	0.3
Lysine	%	0.9
Histidine	%	0.4
Arginine	%	1.0
Tryptophan	%	0.2
*Vitamins*		
Vitamin A	IU/g	15.0
Vitamin D3	IU/g	1.5
Vitamin E	IU/kg	110
Vitamin K3	mg/kg	50
Vitamin B1	mg/kg	17
Vitamin B2	mg/kg	15
Niacin	mg/kg	70
Vitamin B6	mg/kg	18
Pantothenic acid	mg/kg	33
Vitamin B12	mg/kg	0.08
Biotin	mg/kg	0.40
Folate	mg/kg	4
Choline	mg/kg	1200
*Fatty acids*		
C16:0 palmitic	%	0.7
C18 0 stearic	%	0.2
C18:1*ω*9 oleic	%	1.2
C18:2*ω*6 linoeic	%	3.1
C18:3*ω*3 linolenic	%	0.3
Total saturated	%	0.9
Total monounsaturated	%	1.3
Total polyunsaturated	%	3.4
*Supplement*		
Cholesterol	%	0.15
