# Corrigendum to “Caffeoylquinic Acid-Rich Extract of *Aster glehni* F. Schmidt Ameliorates Nonalcoholic Fatty Liver through the Regulation of PPAR*δ* and Adiponectin in ApoE KO Mice”

**DOI:** 10.1155/2019/5317126

**Published:** 2019-10-23

**Authors:** Yong-Jik Lee, Yoo-Na Jang, Yoon-Mi Han, Hyun-Min Kim, Jong-Min Jeong, Min Jeoung Son, Chang Bae Jin, Hyoung Ja Kim, Hong Seog Seo

**Affiliations:** ^1^Cardiovascular Center, Korea University, Guro Hospital, 148 Gurodong-ro, Guro-gu, Seoul 08308, Republic of Korea; ^2^Department of Medical Science, Korea University College of Medicine (BK21 Plus KUMS Graduate Program), Main Building 6F Room 655, 73 Inchon-ro (Anam-dong 5-ga), Seongbuk-gu, Seoul 136-705, Republic of Korea; ^3^Molecular Recognition Research Center, Materials and Life Science Research Division, Korea Institute of Science and Technology, Hwarangno 14 Gil 5, Seoul 136-791, Republic of Korea

In the article titled “Caffeoylquinic Acid-Rich Extract of *Aster glehni* F. Schmidt Ameliorates Nonalcoholic Fatty Liver through the Regulation of PPAR*δ* and Adiponectin in ApoE KO Mice” [1], the Korea University Guro Hospital grant number should be corrected from O1600121 to O1700571. The Acknowledgments section should be corrected as follows.

This research was supported by an intramural grant from the Korea Institute of Science and Technology (2E26990), a grant from the National Research Foundation of Korea (NRF-2016R1A2B3013825), a Korea University grant, a Korea University Guro Hospital Grant (O1700571), and a grant from BK21 Plus Korea University Medical Science Graduate Program. The authors thank Yoon Namkung for proofreading the manuscript and Tae Woo Jung for helping in experimental preparation.
